# *Ziziphus jujube* Fruit Supplementation Improves Liver Enzymes, Lipid Profile, and Hepatic Steatosis but Not Fasting Blood Glucose in Patients With NAFLD: A Randomized Controlled Trial

**DOI:** 10.1002/fsn3.72228

**Published:** 2026-08-10

**Authors:** Ronak Borzooei, Karim Parastouei, Maryam Taghdir, Mojtaba Sepandi, Mohammad Ali Abyazi Heris

**Affiliations:** ^1^ Student Research Committee Baqiyatallah University of Medical Sciences Tehran Iran; ^2^ Health Research Centre, Life Style Institute Baqiyatallah University of Medical Sciences Tehran Iran; ^3^ Department of Nutrition and Food Hygiene, Faculty of Health Baqiyatallah University of Medical Sciences Tehran Iran; ^4^ Baqiyatallah Research Center for Gastroenterology and Liver Diseases (BRCGL) Baqiyatallah University of Medical Sciences Tehran Iran

**Keywords:** hepatic steatosis, non‐alcoholic fatty liver disease, *Ziziphus jujube*

## Abstract

Non‐alcoholic fatty liver disease (NAFLD) is characterized by the excessive accumulation of triglycerides within hepatocytes. *Ziziphus jujube* fruit (ZJF), a medicinal plant with well‐documented antioxidant and anti‐inflammatory properties, has shown hepatoprotective effects in preclinical studies. This study aimed to investigate the effects of ZJF powder supplementation on fasting blood glucose (FBG), lipid profile, liver enzyme levels, and the severity of hepatic steatosis in patients with NAFLD. In this single‐blind, randomized controlled clinical trial, 88 patients diagnosed with NAFLD were recruited. Participants were randomly assigned to either the intervention group, which received 30 g of ZJF powder daily alongside a low‐calorie diet, or the control group, which adhered solely to a low‐calorie diet for 12 weeks. The primary outcome was FBG. Secondary outcomes included lipid profile (total cholesterol [TC], triglycerides [TG], low‐density lipoprotein cholesterol [LDL‐C], and high‐density lipoprotein cholesterol [HDL‐C]), liver enzymes (alanine aminotransferase [ALT], aspartate aminotransferase [AST], and gamma‐glutamyl transpeptidase [GGT]), and the severity of hepatic steatosis (assessed via ultrasonography). Measurements were taken at baseline and post‐intervention. After adjusting for baseline values, weight, and waist circumference using ANCOVA, no significant between‐group difference was observed for the primary outcome, FBG (*p* = 0.12). However, the intervention group demonstrated significantly greater reductions in the secondary outcomes of ALT (*p* = 0.015), AST (*p* = 0.001), LDL‐C (*p* = 0.04), TC (*p* = 0.004), and the severity of hepatic steatosis (*p* = 0.001) compared to the control group. No significant between‐group differences were observed for TG, HDL‐C, or GGT. In this randomized controlled trial, daily supplementation with ZJF powder (30 g/day) in conjunction with a low‐calorie diet for 12 weeks did not significantly reduce FBG (the primary outcome) compared to diet alone. However, significant improvements were observed in secondary outcomes, including ALT, AST, TC, LDL‐C, and most notably hepatic steatosis grade. These findings suggest that ZJF may offer metabolic and hepatoprotective benefits in patients with NAFLD, although the primary outcome remained inconclusive. Larger, longer‐term studies with a placebo control are warranted to confirm these effects.

**Trial Registration:** The protocol of the study was registered at the Iranian Registry of Clinical Trials (IRCT) (IRCT ID: IRCT20140502017522N5; Registration date: June 2023), http://en.irct.ir/trial/69945

## Graphical abstract

Daily supplementation with *Ziziphus jujube* fruit improved liver enzymes, lipid profile, and hepatic steatosis in patients with non‐alcoholic fatty liver disease (NAFLD), potentially through its antioxidant, anti‐inflammatory, and lipid‐modulating properties.

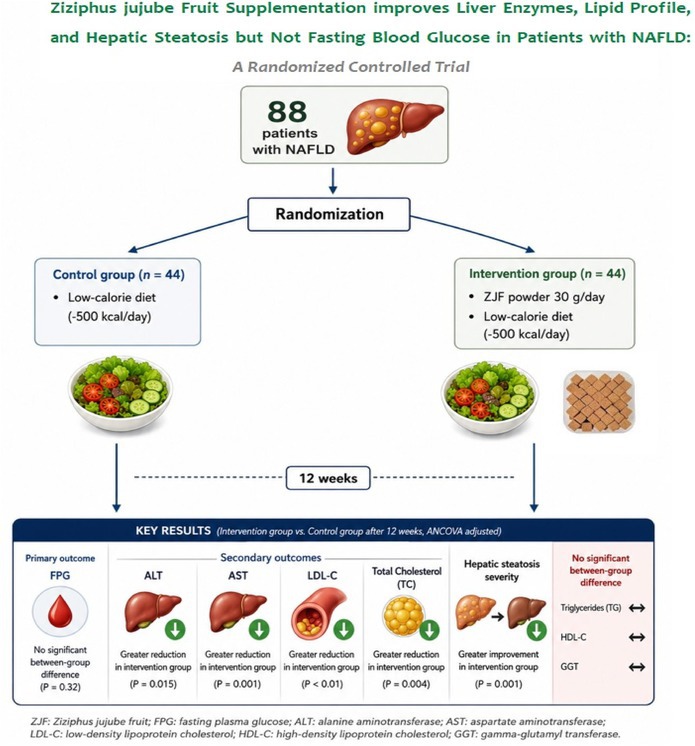

## 1 Introduction

Nonalcoholic fatty liver disease (NAFLD) is a reversible condition characterized by the accumulation of triglycerides in the liver (Perla et al. 2017). NAFLD encompasses a spectrum ranging from simple steatosis to non‐alcoholic steatohepatitis (NASH), which can progress to cirrhosis and hepatocellular carcinoma (Loria et al. 2010). The estimated global prevalence of NAFLD among adults is approximately 32%, with similarly high rates reported in Iran (33%) (Riazi et al. 2022; Tabaeian et al. 2024).

At the time of study design and participant enrollment, the condition was diagnosed as NAFLD based on established criteria. In June 2023, a multisociety Delphi consensus statement recommended replacing NAFLD with the term Metabolic Dysfunction‐Associated Steatotic Liver Disease (MASLD) to reflect the central role of metabolic dysfunction in disease pathogenesis (Rinella et al. 2024). NAFLD is closely associated with metabolic disorders, including type 2 diabetes, obesity, cardiovascular disease, insulin resistance, and hypertriglyceridemia (Xia et al. 2019). Patients with NAFLD are often asymptomatic. Liver enzymes, such as alanine aminotransferase (ALT) and aspartate aminotransferase (AST), can be normal or elevated in NAFLD (Eslami et al. 2013). Although liver biopsy is the gold standard for diagnosing NAFLD, it is invasive and expensive; therefore, ultrasound is widely used (Neuberger and Cain 2021). Triglyceride accumulation in the liver leads to steatohepatitis or fibrosis by increasing susceptibility to damage caused by inflammatory cytokines, mitochondrial dysfunction, and oxidative stress (Mantena et al. 2008). Studies have shown that some herbs can reduce the symptoms of NAFLD by improving lipid metabolism, insulin resistance, inflammation, and oxidative stress (Jayanti et al. 2024).

*Ziziphus jujube* fruit (ZJF) possesses antioxidant, anticancer, and anti‐inflammatory properties due to its polyphenols, alkaloids, terpenoids, polysaccharides, and carotenoids (Eslahi et al. 2018). *Ziziphus jujuba* Mill., commonly known as jujube, Chinese date, or red date, is a species belonging to the family Rhamnaceae (buckthorn family) within the order Rosales. The fruit is an oval drupe, typically 1.5–3.5 cm in length and 1.5–2 cm in diameter, containing a single hard stone (endocarp) with one or two seeds (Gao et al. 2013). The fruiting season in iran typically spans from August to October. Historically, *Z. jujuba* has been extensively used in traditional Eastern medicine for the treatment of various ailments, including liver disorders, insomnia, anemia, diarrhea, diabetic complications, cancer, and loss of appetite (Shahrajabian et al. 2019). The fruit is considered to possess aperient, demulcent, expectorant, and tonic properties. Phytochemical analyses have revealed that jujube fruits are rich in a diverse array of bioactive compounds, including polyphenols, flavonoids, triterpenic acids (such as betulinic acid and lupeol), polysaccharides, vitamin C, amino acids, and dietary fiber. These constituents are responsible for the fruit's wide‐ranging pharmacological effects, which include antioxidant, anti‐inflammatory, hepatoprotective, anticancer, immunostimulating, anti‐obesity, and neuroprotective activities (Bai et al. 2016). These cumulative findings provide a strong scientific rationale for investigating the potential therapeutic effects of *Z. jujuba* fruit in patients with NAFLD (Mohammadifar et al. 2018). Jujube can bind to free radicals and reduce toxic effects and inflammatory responses in the liver (Ji et al. 2017). In one study, taking 30 g/day of jujube for 12 weeks had beneficial effects on lipid profiles and Hemoglobin A1C (HbA1c) (Yazdanpanah et al. 2017). Administration of jujube extract to mice fed a high‐fat diet significantly improved the antioxidant defense system and had hepatoprotective effects (Liu et al. 2017). To the best of our knowledge, there are no studies on the effect of ZJF on metabolic indicators and the severity of hepatic steatosis in patients with NAFLD. To address this gap, we conducted a single‐blind randomized controlled trial to evaluate the effect of ZJF powder on fasting blood glucose (FBG, primary outcome), lipid profile, liver function, and severity of hepatic steatosis (secondary outcomes) in patients with NAFLD. FBG was selected as the primary outcome due to its central role in the metabolic dysregulation underlying NAFLD and because it served as a well‐established endpoint for sample size calculation based on previous ZJF research in metabolic disorders (Sabzghabaee et al. 2013).

## 2 Methods and Materials

### 2.1 Study Design and Participants

This study is a single‐blind controlled clinical trial. Participants were selected from NAFLD patients referred to Hazrat Abolfazl Hospital Clinic in Kermanshah, Iran, from July to December 2023. The inclusion criteria were as follows: diagnosis of NAFLD based on an ultrasound report; age between 18 and 65 years; no alcohol consumption; non‐smoking status; and no use of hepatotoxic medications (tamoxifen, amiodarone, or methotrexate), anti‐glycemic medications, anti‐hyperlipidemia medications, or multivitamin and mineral supplements in the last 3 months.

Participants were diagnosed with NAFLD based on ultrasound evidence of hepatic steatosis, absence of significant alcohol consumption, and exclusion of other liver diseases. This diagnostic approach was consistent with the NAFLD criteria that were standard clinical practice. Upon retrospective review, the majority of participants met at least one cardiometabolic risk factor (obesity, elevated fasting glucose, and/or dyslipidemia) consistent with the newly established MASLD criteria (Rinella et al. 2024).

All participants were referred by a gastroenterology specialist from Hazrat Abolfazl Hospital Clinic. The specialist conducted a thorough clinical evaluation including detailed medical history, physical examination, and review of available laboratory and imaging data—to exclude other etiologies of liver disease, such as hepatitis B and C, autoimmune hepatitis, Wilson's disease, hemochromatosis, alpha‐1‐antitrypsin deficiency, biliary obstruction, and diabetes mellitus. Participants with any of these conditions were not referred to the study. The exclusion criteria included alcohol consumption, pregnancy or lactation, and known or suspected allergy to ZJF. Although *Ziziphus jujuba* is widely consumed as a food, documented cases of IgE‐mediated allergic reactions, including urticaria, angioedema, dyspnea, and anaphylaxis, have been reported in sensitized individuals (Aberumand and Borici‐Mazi 2020). Furthermore, jujube has been implicated in latex‐fruit syndrome due to cross‐reactivity with latex allergens (Lee et al. 2004). Therefore, to ensure participant safety during the 12‐week intervention, individuals with a history of allergic reactions to ZJF or related fruits were excluded.

The sample size was calculated based on a mean difference in FBG of 5.6 mg/dL, with standard deviations of 7.6 mg/dL and 10 mg/dL in the two groups, as reported in a previous study (Sabzghabaee et al. 2013). The calculation assumed a type I error of 0.05, 80% statistical power, and an attrition rate of 10%. Therefore, 88 patients were enrolled in the study (44 participants in each group). Random allocation was performed using the block randomization method (block size = four). The randomization (22 blocks) sequence was created applying a computer‐generated list of random numbers to make a series of sequentially numbered envelopes. The random numbers were located in sealed and opaque envelopes. Thus, eighty‐eight eligible patients were randomly assigned to either the intervention or control groups. The outcome assessor was unaware of the patients' assignment status to the study groups. Participants were not blinded to the intervention due to the nature of the supplement.

All participants provided written informed consent, and the study was approved by the Ethics Committee for Research at Baqiyatallah University of Medical Sciences (IR.BMSU.BAQ.REC.1402.010).

### 2.2 Intervention

The intervention group received 30 g of ZJF powder daily as a snack, alongside a low‐calorie diet (−500 kcal/day), while the control group received only the low‐calorie diet (−500 kcal/day) for 12 weeks. The ZJF powder was manufactured by Shaina Sharq Jujube Company (South Khorasan, Iran). The production process involved drying whole *ZJF* (including the seed), followed by grinding into a fine powder, which was then compressed into cubes (similar in appearance to sugar cubes) without the addition of any preservatives, sweeteners, or other additives. The manufacturer provided a certificate of analysis confirming that the product was prepared from 100% pure *ZJF* with no additives; however, the powder was not standardized to specific bioactive marker compounds (e.g., flavonoids, triterpenic acids, or jujubosides). Each participant was provided with a 12‐week supply of daily pre‐packaged pouches, each containing 30 cubes (totaling 30 g).

An independent Certificate of Analysis (Hamoon Azmay‐e Shargh Laboratory, Iran) confirmed product identity, purity, and absence of contaminants. Proximate analysis of the specific batch used in this study revealed moisture content 4.36%, total ash 2.16%, acid‐insoluble ash 0.02%, and total carbohydrate 46.18% (based on Iranian National Standards; Table S1). The product texture was described as cohesive and uniform per expert assessment. The product was stored in a cool, dry place in sealed, light‐protected containers until distribution to participants. It is important to note that the product was not standardized to specific bioactive marker compounds such as total phenolic content, total flavonoid content, or triterpenoids (e.g., betulinic acid), which represent a limitation of the study.

Participants were instructed to consume the cubes throughout the day, and were advised to divide the daily intake into two or three portions (e.g., 15 cubes in the morning and 15 in the afternoon). To objectively monitor adherence, participants were asked to return all empty daily pouches and any unconsumed cubes at the 6‐ and 12‐week follow‐up visits. The research team counted the remaining cubes to calculate adherence; participants with less than 80% consumption of the total provided cubes were considered non‐adherent, although no participants fell below this threshold during the study. In addition to this objective counting, biweekly telephone interviews were conducted to assess tolerability, side effects, and general compliance. All participants (in both groups) were explicitly instructed at enrollment not to consume any form of *ZJF* (including dried fruit, powder, supplements, tea, or other preparations) outside of the study protocol. During the biweekly telephone interviews and at the 6‐ and 12‐week follow‐up visits, participants were specifically asked about any consumption of jujube or related herbal products, and all participants denied such consumption. Daily energy intake was estimated for each participant by a registered dietitian using the Mifflin–St Jeor equation, based on baseline weight, height, age, sex, and physical activity level.

### 2.3 Laboratory Tests

At baseline and at the end of the 12th week, after 10–12 h of overnight fasting, a 5 mL blood sample was taken from all patients. Serum levels of FBG, lipid profile (total cholesterol [TC], triglycerides [TG], high‐density lipoprotein cholesterol [HDL‐C]), and liver function tests (AST, ALT, and gamma‐glutamyl transpeptidase [GGT]) were measured using enzymatic colorimetric methods with commercial kits (Pars Azmoon Inc., Tehran, Iran). Low‐density lipoprotein cholesterol (LDL‐C) was calculated using Friedewald's formula (Friedewald et al. 1972).

### 2.4 Steatosis Grade Assessment

The status of hepatic steatosis in patients was assessed using ultrasonography with a Mindray DC‐EXP ultrasound machine (Shenzhen Mindray Bio‐Medical Electronics Co. Ltd., Shenzhen, China) at a frequency of 16 MHz, both at baseline and at the end of the 12th week. Hepatic steatosis status was graded categorically into four grades (0, 1, 2, and 3), representing normal, mild, moderate, and severe, respectively, as this device provides qualitative grading rather than quantitative numerical values (Brunt et al. 1999). All sonographic evaluations were performed by a single experienced radiologist who was completely blinded to the participants' treatment allocation. The radiologist was not involved in the randomization process, participant enrollment, or any other aspect of the study conduct, and had no knowledge of group assignment. The use of a single radiologist was intended to reduce inter‐observer variations, as the visual assessment of NAFLD by ultrasonography is known to have substantial interobserver variability. We did not perform formal intra‐observer or inter‐observer reliability assessments (e.g., Cohen's κ) due to practical constraints; however, the use of a single blinded radiologist with standardized grading criteria was intended to ensure consistency and minimize measurement bias.

### 2.5 Anthropometric Assessment

Weight, height, and waist circumference (WC) of all participants were measured at baseline and at the end of the 12th week. Weight was measured with an accuracy of 100 g, with minimal clothing and without shoes. Height was measured using a tape measure to the nearest 0.5 cm. Waist circumference was measured at the narrowest area between the last rib and the iliac crest in a standing position using a non‐elastic tape. Body mass index (BMI) was calculated as weight (kg) divided by the square of height (m^2^).

### 2.6 Dietary and Physical Activity Assessment

Dietary intake was assessed using three 24‐h dietary recalls (including two working days and one weekend day) administered by a trained dietitian at both baseline and the end of the 12th week. This method captures detailed information on all foods, beverages, and dietary supplements consumed during the preceding 24‐h period. Nutritional data were analyzed using Nutritionist IV software (First Databank Inc., San Bruno, CA, USA) to calculate daily energy, macronutrient, and micronutrient intakes. Participants were not provided with specific meal plans; instead, they received general dietary counseling on how to achieve a 500 kcal/day deficit through a balanced diet (45%–65% carbohydrate, 15%–20% protein, 25%–35% fat). Additionally, all participants in both groups were advised to perform 30–45 min of daily physical activity as part of the comprehensive lifestyle modification approach. This recommendation was provided by the registered dietitian during the initial counseling session and reinforced during biweekly telephone interviews. Physical activity levels were measured using the International Physical Activity Questionnaire (IPAQ) at both baseline and the end of the 12th week to quantify actual changes in physical activity (Craig et al. 2003).

### 2.7 Statistical Analysis

All data were analyzed using SPSS Version 24 (SPSS Inc., Chicago, IL). The normality of the variables was assessed using the Kolmogorov–Smirnov test. Data are expressed as mean ± standard deviation (SD) for continuous variables and as *n* (%) for categorical variables. The Chi‐square test, two independent samples *t*‐test, and paired *t*‐test were used for univariate analysis. Analysis of covariance (ANCOVA) was used to compare the differences in post‐treatment values of parameters, adjusting for baseline values of the dependent variables and covariates (weight and WC). Variables considered potential confounders included baseline values of the dependent variables, weight, and WC, which differed significantly between groups at baseline. These variables were included as covariates in the ANCOVA models. We did not adjust for age, sex, physical activity, or dietary intake because these variables did not differ significantly between groups at baseline and were therefore not considered confounders. *p*‐Values < 0.05 were considered statistically significant.

## 3 Results

**FIGURE 1:**
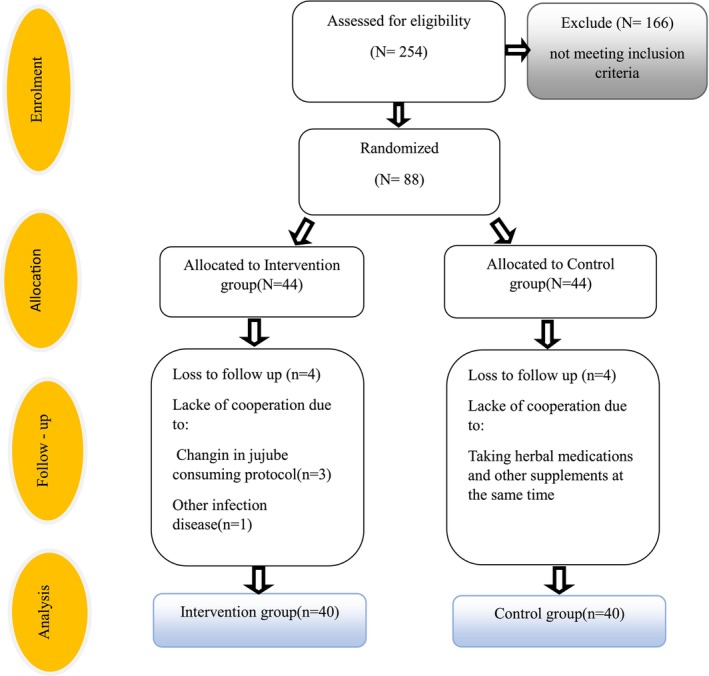
Flow chart of the study participants.

A total of 254 patients were assessed for eligibility, and finally, 88 eligible participants were enrolled. Eighty participants [intervention group (*n* = 40) and control group (*n* = 40)] completed the study (Figure 1). No harm or side effects including allergic reactions were reported or observed throughout the 12‐week intervention period.

As shown in Table 1, baseline and demographic data revealed significant differences between the ZJF and control groups regarding weight (*p* = 0.018), BMI (*p* = 0.007), and WC (*p* = 0.033).

**TABLE 1:** Baseline and demographic characteristics of patients in the *Ziziphus jujube* group and the control group.

Variable	Intervention group (*n* = 40)	Control group (*n* = 40)	*p* ^a^
Age (years)	49.10 (13.64)	43.60 (13.04)	0.069
Gender			0.17
Male, *n* (%)	26 (65)	20 (50)	
Female, *n* (%)	14 (35)	20 (50)	
Steatosis grade, *n* (%)			0.13
Grade 1	11 (27.5)	6 (15)	
Grade 2	25 (62.5)	24 (60)	
Grade 3	4 (10)	10 (25)	
Height (cm)	164.29 (7.93)	165.02 (8.44)	0.539
Weight (kg)	82.85 (13.83)	91.85 (19.00)	0.018
BMI (kg/m^2^)	28.07 (5.17)	31.74 (6.59)	0.007
WC (cm)	96.20 (11.49)	103.100 (16.47)	0.033

*Note:* Results are presented as mean ± SD.

Abbreviations: BMI, body mass index; WC, waist circumferences.

^a^ Independent two‐sample *t*‐test.

As shown in Table 2, both groups achieved significant within‐group reductions in energy, carbohydrate, protein, and fat intake over the 12‐week study period (all *p* < 0.05). Specifically, the intervention group reduced energy intake by 360.3 kcal/day (from 2373.9 to 2013.6 kcal/day), while the control group reduced energy intake by 379.7 kcal/day (from 2398.3 to 2018.6 kcal/day). These reductions were consistent with the prescribed −500 kcal/day deficit, accounting for expected underreporting in dietary recall data. Importantly, there were no statistically significant between‐group differences in any dietary component or physical activity level at baseline or week 12 (all *p* > 0.05 for between‐group comparisons), confirming that both groups adhered equally well to the dietary recommendations. Therefore, the observed between‐group differences in anthropometric and biochemical outcomes are unlikely to be confounded by differential changes in dietary intake or physical activity.

**TABLE 2:** Dietary intakes and physical activity in the *Ziziphus jujube* group and the control group during the period of study.

Variable	Intervention group (*n* = 40)	Control group (*n* = 40)	*p* ^a^
Energy (kcal)			
Before	2373.99 (249.29)	2398.27 (236.65)	0.65
After	2013.653 (212.96)	2018.57 (205.88)	0.92
*p* ^b^	0.001	0.001	
Carbohydrate (g/d)			
Before	370.29 (66.59)	356.00 (64.26)	0.33
After	297.60 (59.02)	282.71 (53.02)	0.23
*p* ^b^	0.001	0.001	
Protein (g/d)			
Before	73.18 (21.57)	77.42 (17.98)	0.34
After	67.61 (20.18)	68.11 (21.48)	0.91
*p* ^b^	0.002	0.001	
Fat (g/d)			
Before	88.23 (14.89)	88.67 (17.34)	0.90
After	73.28 (13.90)	73.69 (18.22)	0.91
*p* ^b^	0.001	0.001	
Physical activity (MET‐h/week)			
Before	31.97 (2.28)	32.75 (1.79)	0.09
After	32.65 (2.26)	33.057 (1.70)	0.37
*p* ^b^	< 0.001	0.03	

*Note:* Results are presented as mean ± SD.

^a^ Between‐group *p*‐values were calculated using independent *t*‐tests and indicate differences between the two groups at each time point (baseline and week 12).

^b^ Within‐group *p*‐values were calculated using paired *t*‐tests and indicate significant changes from baseline to week 12 within each group.

**FIGURE 2:**
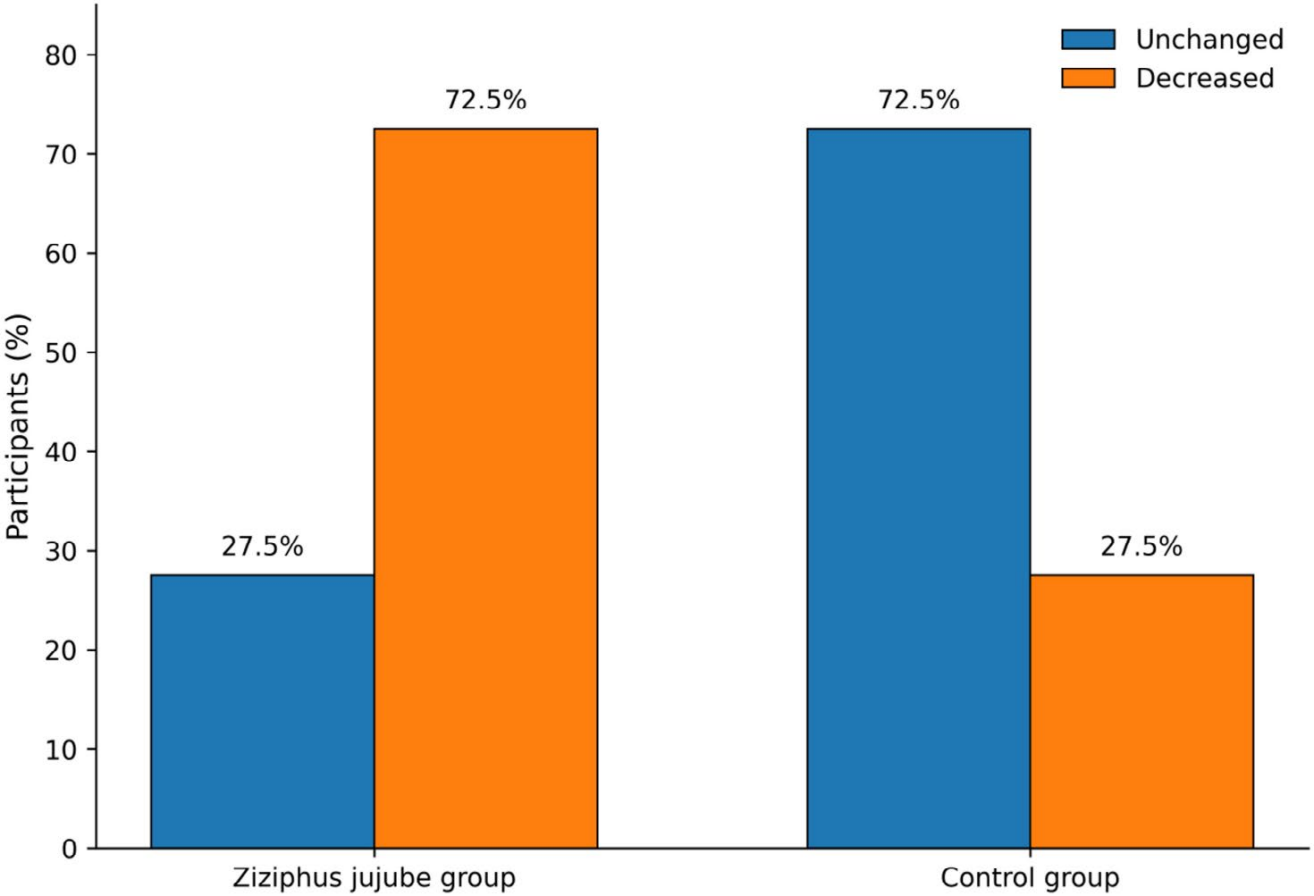
Change in hepatic steatosis grade after 12 weeks.

Table 3 presents the anthropometric and biochemical indices in the ZJF and control groups during the study. After adjusting for baseline values, weight, and WC, the ZJF group demonstrated significantly greater reductions in ALT (adjusted mean difference: −4.58 U/L, 95% CI: −8.24 to −0.92; *p* = 0.015; Cohen's *d* = 0.25), AST (−5.59 U/L, 95% CI: −8.44 to −2.75; *p* < 0.001; Cohen's d = 0.33), LDL‐C (−5.89 mg/dL, 95% CI: −11.56 to −0.23; *p* = 0.04; Cohen's *d* = 0.20), and TC (−13.02 mg/dL, 95% CI: −21.72 to −4.34; *p* = 0.004; Cohen's d = 0.36) compared to the control group. The magnitude of these effects ranged from small‐to‐moderate, with the TC reduction approaching a moderate effect size. No significant between‐group differences were observed for FBG, TG, HDL‐C, or GGT. Furthermore, the severity of hepatic steatosis decreased in 29 patients (72.5%) in the ZJF group compared to 11 patients (27.5%) in the control group (*p* = 0.001), representing a clinically large and meaningful improvement (Figure 2).

**TABLE 3:** Anthropometric and biochemical indices in the *Ziziphus jujube* group and the control group during the period of study.

Variable	Intervention group (*n* = 40)	Control group (*n* = 40)	*p* ^a^	Adjusted mean difference (95% CI)	*p* ^c^
Weight (kg)					
Before	82.85 (13.83)	91.85 (19.00)	**0.018**		
After	80.85 (12.82)	89.30 (17.77)	**0.017**	−0.16 (−1.16, 0.83)	0.74
*p* ^b^	**< 0.001**	**< 0.001**			
BMI (kg/m^2^)					
Before	28.07 (5.17)	31.74 (6.59)	**0.007**		
After	27.40 (4.79)	30.71 (6.01)	**0.008**	0.01 (−0.38, 0.41)	0.94
*p* ^b^	**< 0.001**	**< 0.001**			
WC (cm)					
Before	96.20 (11.49)	103.100 (16.47)	**0.033**		
After	94.68 (10.65)	101.25 (15.31)	**0.029**	−0.25 (−1.17, 0.65)	0.57
*p* ^b^	**< 0.001**	**< 0.001**			
FBG (mg/dL)					
Before	101.47 (12.83)	104.32 (18.45)	0.425		
After	96.82 (11.91)	96.45 (11.12)	0.885	2.95 (−0.86, 6.77)	0.12
*p* ^b^	**0.028**	**< 0.001**			
TG (mg/dL)					
Before	194.12 (78.83)	244.37 (102.97)	**0.017**		
After	145.52 (59.76)	187.70 (81.15)	**0.01**	−8.06 (−28.81, 12.69)	0.44
*p* ^b^	**< 0.001**	**< 0.001**			
TC (mg/dL)					
Before	211.97 (32.61)	202.22 (41.52)	0.246		
After	190.12 (31.88)	195.85 (36.31)	0.456	−13.02 (−21.72, −4.34)	**0.004**
*p* ^b^	**< 0.001**	**0.006**			
LDL‐c (mg/dL)					
Before	114.7 (25.51)	108.02 (34.03)	0.324		
After	105.80 (27.92)	104.62 (33.00)	0.864	−5.89 (−11.56, −0.23)	**0.04**
*p* ^b^	**< 0.001**	**< 0.001**			
HDL‐c (mg/dL)					
Before	41.67 (10.01)	42.00 (12.25)	0.897		
After	43.72 (10.16)	42.55 (11.19)	0.625	1.36 (−0.17, 2.89)	0.08
*p* ^b^	**0.004**	0.108			
GGT (U/L)					
Before	23.20 (6.28)	31.05 (4.26)	0.238		
After	20.09 (6.01)	30.24 (6.92)	**0.047**	−0.52 (−1.37, 0.32)	0.22
*p* ^b^	**0.049**	0.051			
ALT (U/L)					
Before	34.22 (18.64)	37.80 (20.82)	0.421		
After	27.60 (17.48)	34.87 (17.29)	0.065	−4.58 (−8.24, −0.92)	**0.015**
*p* ^b^	**< 0.001**	**< 0.001**			
AST (U/L)					
Before	32.47 (16.94)	37.55 (17.64)	0.193		
After	25.62 (10.84)	34.80 (15.73)	**0.003**	−5.59 (−8.44, −2.75)	**< 0.001**
*p* ^b^	**< 0.001**	**< 0.001**			

*Note:* Data are presented as mean ± standard deviation. Significant findings are in bold.

Abbreviations: ALT: alanine transaminase; AST: aspartate transaminase; BMI: body mass index; FBG: fasting blood glucose; GGT: gamma‐glutamyl transferase; HDL: high‐density lipoprotein; LDL: low‐density lipoprotein; TC: total Cholesterol; TG: triglyceride; WC: waist circumferences.

^a^ Independent two‐sample *t*‐test.

^b^ Paired sample *t*‐test.

^c^ ANCOVA test, adjusted for weight and WC.

## 4 Discussion

This study investigated the effect of ZJF powder on FBG, lipid profile, liver function, and the severity of hepatic steatosis in patients with NAFLD. Both groups followed a low‐calorie diet (−500 kcal), which led to significant reductions in FBG, TG, TC, LDL‐C, ALT, and AST by the end of the study. However, only the ZJF group experienced a significant decrease in GGT and a significant increase in HDL‐C. After adjusting for confounding variables, the results indicated that ZJF powder at a dose of 30 g/day for 12 weeks, along with a low‐calorie diet, may have beneficial effects on ALT, AST, TC, LDL‐C, and the severity of hepatic steatosis in patients with NAFLD. However, no significant between‐group differences were observed in FBG, HDL‐C, and TG.

The low‐calorie diet (−500 kcal/day) was selected as the background intervention based on current clinical guidelines, which recommend lifestyle modification particularly caloric restriction and weight loss as the cornerstone of NAFLD management (Pouwels et al. 2022). Caloric restriction independently improves insulin sensitivity (Pardo et al. 2019), reduces systemic and hepatic inflammation (Larson‐Meyer et al. 2008), and decreases oxidative stress (Galassetti et al. 2006). Ethically, withholding standard dietary recommendations from any participant would be inappropriate, and providing both groups with the same dietary intervention allowed us to isolate the specific effect of ZJF supplementation above and beyond standard care. Both groups demonstrated significant reductions in energy, carbohydrate, protein, and fat intake (Table 2), with no significant between‐group differences in dietary intake or physical activity at baseline or week 12, indicating comparable adherence. Despite this, the ZJF group showed significantly greater improvements in ALT, AST, TC, LDL‐C, and hepatic steatosis grade compared to the control group, suggesting that ZJF provides additional hepatoprotective benefits beyond those achieved by caloric restriction alone.

Specifically, after adjusting for confounding variables, ZJF powder significantly reduced TC and LDL‐C levels in the intervention group compared with the control group. However, no significant differences were observed in FBG, HDL‐C, TG, and GGT. Irannejad Niri et al. (2021) reported that consumption of 30 g/day of dried *Ziziphus vulgaris* fruit did not significantly change FBG, TC, LDL‐C, HDL‐C, AST, and ALT in patients with type 2 diabetes (Irannejad Niri et al. 2021). In another study, it was found that 30 g/day of *Ziziphus jujube* for 12 weeks significantly reduced FPG, TG, TC, and LDL‐C in patients with type 2 diabetes (Farhadnejad et al. 2022). Yazdanpanah et al. (2017) observed significant reductions in TC, TG, and LDL‐C following 12 weeks of 10 g/day of ZJF infusion intake in patients with type 2 diabetes (Yazdanpanah et al. 2017). Another study showed that daily intake of 15 g of ZJF powder had a significant effect on TC and LDL‐C in obese adolescents (Sabzghabaee et al. 2013). Researches suggest that ZJF extract may reduce serum lipids through multiple mechanisms. ZJF contains pectin, saponins, inulin, betulinic acid, and phytosterols that can inhibit cholesterol digestion and absorption (Choi et al. 2011). ZJF extract likely reduces serum lipids by increasing cholesterol and bile acid excretion and inhibiting cholesterol and lipid synthesis enzymes (Jurgoński et al. 2014; Liu et al. 2016). Due to its phenolic compounds, ZJF may play a role in lipolysis and lipid oxidation by modulating the expression of peroxisome proliferator‐activated receptor gamma (PPARγ) and lipoprotein lipase genes (Savova et al. 2021). ZJF polyphenols can lead to reduced fat absorption by inhibiting pancreatic phospholipase (Trifonova et al. 2021). Betulinic acid can reduce lipid accumulation in liver cells and adipocytes by inhibiting lipogenesis and enhancing β‐oxidation. Betulinic acid activates AMP‐activated protein kinase (AMPK) and reduces the expression of lipogenic enzymes such as acetyl‐CoA carboxylase, fatty acid synthase, and stearoyl‐CoA desaturase (Kim and Go 2017; Mohsen et al. 2019; Quan et al. 2013).

The significant reductions in LDL‐C and TC observed in the intervention group are consistent with multiple mechanistic pathways. ZJF contains bioactive compounds including pectin, phytosterols, and betulinic acid that inhibit cholesterol absorption, suppress HMG‐CoA reductase activity, and activate AMPK, leading to reduced cholesterol synthesis and enhanced LDL clearance. Additionally, modulation of the gut microbiome may contribute to the lipid‐lowering effects of jujube products. In contrast, the lack of significant between‐group differences in TG and HDL‐C may be explained by several factors. First, the substantial within‐group TG reductions in both groups reflecting the potent effect of caloric restriction and weight loss on TG metabolism may have masked any additional effect of ZJF. Second, the high baseline variability in TG levels, particularly in the control group, may have reduced statistical power. Third, HDL‐C is known to be slow to respond to short‐term dietary interventions, and the 12‐week period may have been insufficient to detect a significant change, particularly in the context of active weight loss.

The findings of this study indicated that ZJF powder significantly decreased ALT and AST levels in the ZJF group compared with the control group. The improving effect of ZJF powder on hepatic steatosis was also assessed in this study. Ultrasound results revealed that the grade of hepatic steatosis was reduced in 72.5% of patients in the ZJF group compared to 27.5% in the control group. This significant reduction in hepatic steatosis is aligning with the results of previous studies (Jayanti et al. 2024; Zhang et al. 2024).

The absence of a significant between‐group difference in GGT levels warrants discussion. GGT is a less specific marker of hepatocellular injury than ALT and AST. Moreover, GGT has been reported to be less responsive to short‐term dietary and lifestyle interventions compared to other liver enzymes (Thomas et al. 2006; Volynets et al. 2013). The comparable dietary and physical activity interventions received by both groups may have driven GGT changes in a similar manner, obscuring any additional effect of ZJF. Additionally, the 12‐week intervention period may have been insufficient for GGT to show significant change. Importantly, the lack of GGT improvement does not diminish the clinically meaningful benefits observed in ALT, AST, lipid profile, and hepatic steatosis grade, which are more directly relevant to NAFLD severity and progression.

**FIGURE 3:**
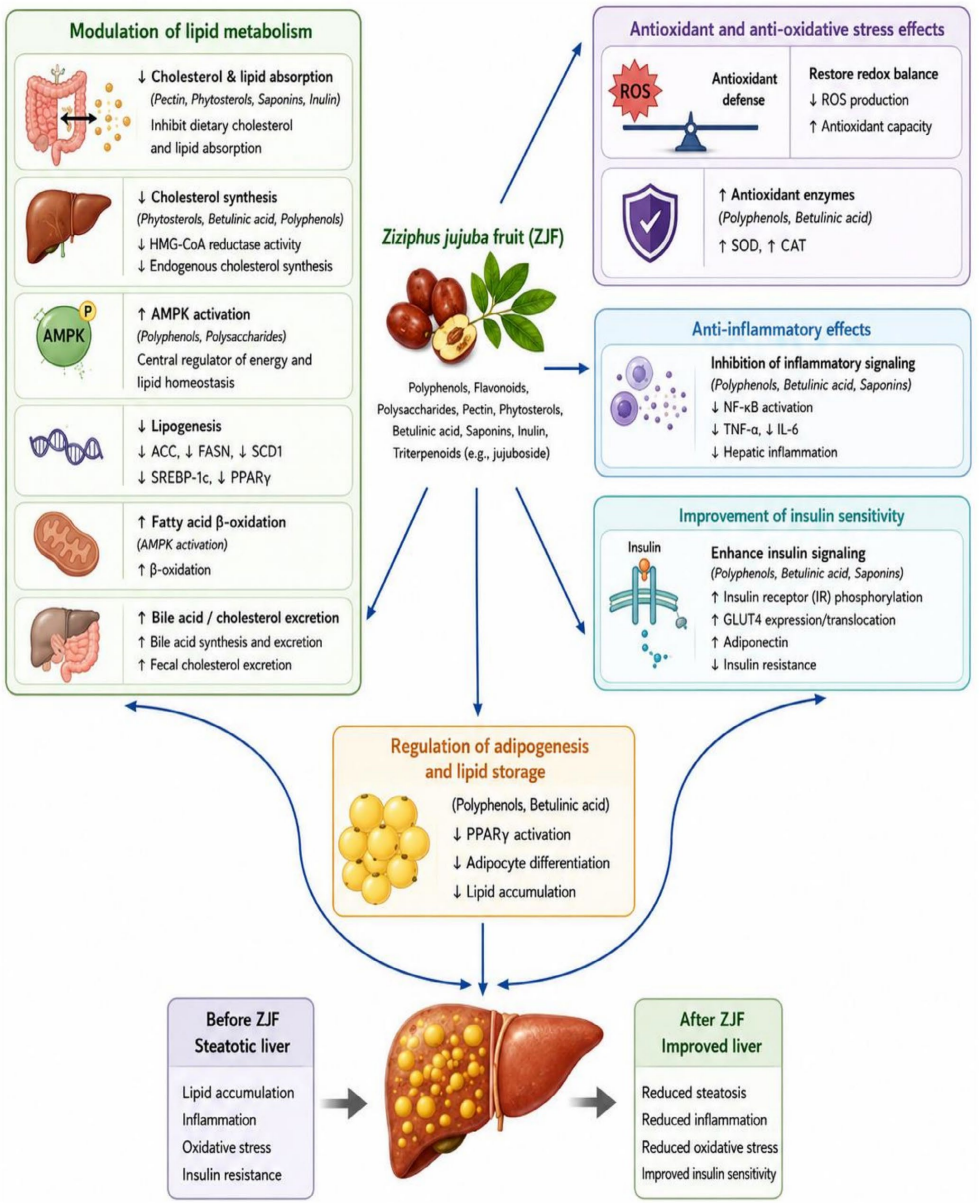
Schematic mechanisms of the effects of ZJF.

Elevated levels of liver enzymes, including AST and ALT, are indicators of hepatocellular injury and liver dysfunction (Jain et al. 2023). Some studies have demonstrated the hepatoprotective effects of *Ziziphus jujube* against various liver injuries. It has been shown that ZJF extract significantly reduced AST and ALT in liver injury (Huang et al. 2017; Kandimalla et al. 2016; Liu et al. 2022; Yu et al. 2020). A study has indicated that *Ziziphus jujuba* Mill. root bark decreased oxidative stress, inflammatory markers, AST and ALT levels in CCl4‐induced liver damage in rats (Kandimalla et al. 2016). Some research suggests several potential pathways through which ZJF may exert its effects. ZJF extracts increase the expression of antioxidant enzymes, including Superoxide dismutase (SOD) and Catalase (CAT), which help reduce hepatic steatosis (Shen et al. 2009). Studies have demonstrated that jujube extracts can reduce the expression of genes involved in lipid metabolism, such as PPARγ, sterol regulatory element‐binding transcription factor 1 (SREBP‐1c), and 3‐hydroxy‐3‐methyl‐glutaryl‐coenzyme A reductase (HMG‐CoA reductase), leading to decreased cholesterol synthesis and inhibition of adipogenesis in adipocytes (Brusotti et al. 2017; Kubota et al. 2009). Another mechanism by which jujube may affect hepatic steatosis is through its triterpenoids, such as lupeol. Lupeol reduces hepatic inflammatory damage and prevents hepatic steatosis by inhibiting the nuclear factor kappa‐light‐chain‐enhancer of activated B cells (NF‐κB) pathway and reducing pro‐inflammatory cytokines such as tumor necrosis factor‐alpha (TNF‐α) and interleukin‐6 (IL‐6) (Daniel‐E‐mail et al. 2023). Jujube can also improve hepatic steatosis by increasing insulin sensitivity through increased expression of the insulin receptor (IR) and glucose transporter type 4 (GLUT4) (Kottireddy and Koora 2019). Additionally, jujube may indirectly reduce lipid accumulation in the liver by increasing adiponectin levels (Hemmati et al. 2015). Figure 3 schematically illustrates the potential mechanisms underlying the effects of *Ziziphus jujube*.

To our knowledge, this study was the first to investigate the effect of ZJF on metabolic indicators, liver function, and the severity of hepatic steatosis in patients with NAFLD. Hazrat Abolfazl Hospital is a reference hospital in Kermanshah, so the results of this study may be representative of patients seeking care for NAFLD in this region of Iran, though multicenter studies are needed for broader generalizability.

Although FBG was designated as the primary outcome, the study had limited power to detect the observed small between‐group difference (2.95 mg/dL; 95% CI: −0.86 to +6.77). The wide confidence interval that includes both no effect and a potentially meaningful increase suggests that the FBG findings are inconclusive, and a larger study would be needed to determine whether ZJF has a meaningful effect on FBG in this population. Importantly, despite the null FBG finding, the study demonstrated significant improvements in multiple clinically relevant secondary outcomes including ALT, AST, LDL‐C, TC, and hepatic steatosis grade for the improvement in steatosis grade. These findings strongly support the therapeutic potential of ZJF in NAFLD.

Several methodological limitations should be acknowledged. First, despite random allocation, there were significant baseline differences in weight, BMI, and WC between the two groups. Although we adjusted for these variables in our statistical models using ANCOVA which is considered an appropriate method for addressing baseline imbalances in RCTs, residual confounding cannot be entirely ruled out. Future studies with larger sample sizes are less likely to experience such imbalances. Second, the single‐blind design and absence of a placebo introduce potential for performance bias and expectation effects, although objective outcomes and comparable lifestyle adherence between groups partially mitigate this concern. Third, the 12‐week intervention period was insufficient to assess long‐term sustainability or hard clinical endpoints. Fourth, while ultrasound was performed by a single blinded radiologist, we did not conduct formal reliability assessments, and ultrasonography has lower sensitivity than MRI‐PDFF or CAP. Fifth, although the sex distribution did not differ significantly between groups (*p* = 0.17), the intervention group had a higher proportion of male participants. Sex‐stratified sensitivity analyses revealed similar treatment effects in males and females, although subgroup sample sizes limited statistical power. Sixth, we acknowledge that the ZJF product used in this study was not standardized to specific bioactive marker compounds (e.g., total phenolic content, total flavonoid content, or triterpenoids such as betulinic acid). While proximate analysis confirmed product purity and consistency (Table S1), the lack of phytochemical standardization limits the ability to directly compare our findings with other studies using different ZJF preparations. The phytochemical composition of *Ziziphus jujuba* is known to vary considerably depending on cultivar, geographical origin, growing conditions, harvesting time, and processing methods (Azami Movahed et al. 2024; Rajaei et al. 2021). Future studies should employ chemically standardized extracts with quantified bioactive compounds to enable more precise dose–response evaluations and facilitate comparability across studies. Finally, the exclusion of other liver diseases was based on clinical judgment rather than systematic laboratory testing, which, while reflecting standard clinical practice, may be considered a limitation. Future double‐blind, placebo‐controlled studies with longer follow‐up, objective imaging techniques, standardized extracts, and larger sample sizes are warranted to confirm our findings.

In conclusion, this randomized controlled trial demonstrated that daily supplementation with 30 g of ZJF powder for 12 weeks, in conjunction with a low‐calorie diet, did not significantly reduce fasting blood glucose (the primary outcome) compared to diet alone. However, significant improvements were observed in secondary outcomes, including ALT, AST, total cholesterol, LDL‐C, and most importantly the severity of hepatic steatosis. The lack of effect on FBG may reflect the relatively short intervention duration, the small effect size, or the potent effect of the low‐calorie diet itself in both groups. The significant improvements in multiple clinically relevant secondary outcomes particularly hepatic steatosis grade, which is the most disease‐specific endpoint in NAFLD suggest that ZJF may offer additional metabolic and hepatoprotective benefits beyond those achieved by dietary modification alone. Given the single‐blind design and absence of a placebo, these findings should be interpreted with caution. Larger, double‐blind, placebo‐controlled trials with longer follow‐up periods and objective imaging techniques (e.g., MRI‐PDFF or CAP) are warranted to confirm these effects and to further elucidate the mechanisms of action.

## Author Contributions

**Ronak Borzooei:** conceptualization, formal analysis, investigation, methodology, writing – original draft, writing – review and editing. **Mohammad Ali Abyazi Heris:** investigation, resources, writing – review and editing. **Karim Parastouei:** data curation, writing – review and editing. **Mojtaba Sepandi:** formal analysis, writing – review and editing, methodology. **Maryam Taghdir:** conceptualization, methodology, formal analysis, supervision, project administration, writing – review and editing.

## Funding

The authors have nothing to report.

## Ethics Statement

The study was approved by the ethics committee for Research at Baqiyatallah University of Medical Sciences (IR.BMSU.BAQ.REC.1402.010).

## Consent

An informed consent form was read and signed by all participants.

## Conflicts of Interest

The authors declare no conflicts of interest.

## Supporting information


**Table S1:** Product characterization of *Ziziphus jujuba* fruit powder used in the study.

## Data Availability

The datasets used and/or analyzed during the current study are available from the corresponding author on reasonable request.
