# Supplementary material for: Ziziphus jujube Fruit Supplementation Improves Liver Enzymes, Lipid Profile, and Hepatic Steatosis but Not Fasting Blood Glucose in Patients With NAFLD: A Randomized Controlled Trial
Source: Food Sci Nutr. 2026 Aug 10;14(8):e72228. doi: 10.1002/fsn3.72228 (PMC13455679; doi:10.1002/fsn3.72228)
Supplement: Supplementary file 1 [file FSN3-14-e72228-s001.docx]

**Supplementary Table S1**

**Supplementary Table S1: Product Characterization of *Ziziphus jujuba* Fruit Powder Used in the Study**

| Parameter | Result | Method |
| --- | --- | --- |
| **Product Information** |  |  |
| Product Name | Ziziphus jujuba Fruit Powder (Compressed Cubes) | — |
| Product Type | Jujube Cubes (*"Habb-e-Anab"*) | — |
| Texture | Cohesive and uniform | Expert assessment |
| **Proximate Analysis** |  |  |
| Moisture Content | 4.36% (w/w) | ISIRI 2705 |
| Total Ash | 2.16% (w/w) | ISIRI 1197 |
| Acid-Insoluble Ash | 0.02% (w/w) | ISIRI 1253 |
| Total Carbohydrate | 46.18% (w/w) | ISIRI 2302 |
| **Phytochemical Composition** | **(Not Available)** | — |
| Total Phenolic Content | Not quantified | — |
| Total Flavonoid Content | Not quantified | — |
| Betulinic Acid | Not quantified | — |
| **Additives** |  |  |
| Preservatives, Sweeteners, or Additives | None | Manufacturer confirmation |
